# Oral Health Advocacy Education Impacts Future Engagement: Exploration at a Midwestern US Dental School

**DOI:** 10.3389/froh.2021.714199

**Published:** 2021-10-06

**Authors:** Homa Amini, Alexander J. Wells, James R. Boynton, Xiaohan Guo, Ai Ni

**Affiliations:** ^1^Division of Pediatric Dentistry, The Ohio State University College of Dentistry, Columbus, OH, United States; ^2^Section of Pediatric Dentistry, Nationwide Children's Hospital, Columbus, OH, United States; ^3^Department of Orthodontics and Pediatric Dentistry, University of Michigan School of Dentistry, Ann Arbor, MI, United States; ^4^Division of Biostatistics, the Ohio State University College of Public Health, Columbus, OH, United States

**Keywords:** dental education, attitudes, advocacy, public policy, curriculum

## Abstract

**Purpose:** With the emergence of COVID-19, and the potential inclusion of dental benefits in Medicare, it is critical that dentists are able to engage in legislative advocacy to support public oral health. Dental education has an opportunity to teach advocacy skills to future dentists, although advocacy training in predoctoral dental education has been largely ignored. The purpose of this study was to evaluate fourth-year dental student's attitudes toward advocacy, identify the type and extent of advocacy experiences during dental school, and assess their future intentions to engage in advocacy.

**Methods:** An electronic questionnaire was administered to fourth-year dental students enrolled in their final semester at Ohio State University.

**Results:** Forty-seven students completed the survey (43% response rate). Most (84%) respondents agreed that advocacy training should be a required experience in accreditation standards for predoctoral dental education. Over half (58%) reported seldom or no exposure to legislative and regulatory processes in oral health policy development in the curriculum. Students who participated in grassroots advocacy efforts while in dental school were more likely to contact legislators regarding dental issues (*p* = 0.005) or public insurance (*p* = 0.037), and participate in future lobbying efforts (*p* = 0.019). Students who contributed to PAC while in dental school were more likely to express intentions to contribute in future (*p* = 0.005).

**Conclusions:** There is limited exposure to legislative advocacy in predoctoral dental education. Dental students with advocacy experience are more likely to report intentions to participate in advocacy as dentists. Dental education has a critical role in preparing future dentist-advocates.

## Introduction

Physicians have long recognized their roles as advocates. Twenty years ago, the American Medical Association adopted a declaration of professional responsibility that called on all physicians to “advocate for social, economic, educational, and political changes that ameliorate suffering and contribute to human well-being” [1, 2]. Today, health advocacy is deemed an essential component of medical practice, and the Accreditation Council for Graduate Medical Education in the US and the Royal College of Physicians and Surgeons in Canada recognize training in advocacy as a critical component of graduate medical education [2, 3].

Advocacy is a broad term and may be defined as efforts at multiple levels to improve quality of life for individuals, families, or communities [3, 4]. Although there is no clear definition of what constitutes advocacy training, community-based block rotations, cultural-immersion experiences, inter-professional rotations, volunteer service opportunity at community-based organization, cultural competency training, training in basic public health principles/social determinants of health, training in public policy, and legislative experiences are considered examples of advocacy training in medical education [4–7].

Advocacy training as part of formal dental education has recently gained some momentum in the United States. In 2013, the American Dental Association's (ADA) Commission on Dental Accreditation (CODA) recognized the importance of health advocacy by pediatric dentists and incorporated advocacy training into educational standards of Advanced Education in Pediatric Dentistry [7]. The standard calls for didactic and clinical training because “pediatric dentists serve as the primary advocates for the oral health of children in America” [8]; therefore, residents need to be adequately trained to assume this role competently. Pediatric dentistry specialty training must provide residents with opportunities for legislative advocacy and participation in organized dentistry to represent the oral health needs of children, particularly the underserved [8].

For adult and elderly populations, general dentists are critically important oral health advocates. According to the ADA, there are approximately 160,000 general dental practitioners in the US [9]. Although the ADA has a strong legislative presence at the national level, individual dentists' grassroots activities play an important role in advancing oral health-related policies. Advocacy is an essential skillset for dental providers in order to promote oral health through legislative, regulatory, and public health policy efforts [10]. Dentists can build their advocacy skills through training and experiences during their formal dental education. However, CODA currently has no specific language related to public policy/advocacy training as part of predoctoral dental education [10, 11].

There are few studies in the literature regarding advocacy training in predoctoral dental education and factors that may influence future engagement in advocacy. Therefore, the purpose of this study was to evaluate fourth-year dental student's attitudes toward advocacy, identify the type and extent of advocacy experiences during dental school, and assess their future intentions to engage in advocacy, particularly legislative advocacy, post-graduation.

## Materials and Methods

This study was reviewed and deemed exempt from IRB review by the Ohio State University Office of Responsible Research Practices (Study ID: 2019E0158). A 46-item questionnaire was developed based on literature review and adapted from previously published advocacy survey instruments [4, 12]. The survey queried respondents several domains including: (1) attitudes toward advocacy and training; (2) exposure to advocacy topics as part of dental education; (3) personal participation in advocacy activities; and (4) intentions for future advocacy involvement post-graduation.

For the purpose of this study, advocacy was defined for fourth-year dental students as “a course of action that involves determination of needs and development of strategies to meet them” [13, 14]. An advocate was defined as “one who speaks up, pleads for, or champions a cause while applying professional expertise and leadership to support efforts on individual (family or patient), community, and legislative/policy levels, which result in the improved quality of life for individuals, families, or communities” [6].

The survey was distributed electronically via SurveyMonkey (SurveyMonkey Inc., San Mateo, California, USA) to 109 fourth-year dental students enrolled in their final semester at The Ohio State University (OSU) College of Dentistry. The response period was 2 months, between April and June of 2019, with three reminders sent during that time.

Descriptive statistics were used to characterize attitudes toward advocacy training and describe the extent and spectrum of activities students engaged in. Not all respondents answered all questions, and percentages were calculated based on the total that responded to each question. Logistic modeling was used to test for certain associations regarding student's experiences and future intentions. Wald test was used to test the regression coefficients against zero. Analysis was conducted using R Statistical Software version 4.0.2 (R Foundation for Statistical Computing, Vienna, Austria) [15]. A *P*-value ≤ 0.05 was considered significant.

## Results

Out of 109 fourth-year dental students, forty-seven completed the survey, yielding a 43% response rate. Over half (57%) of the respondents were male which was relatively similar to the overall class gender distribution (63% male). Most (96%) respondents agreed or strongly agreed that advocacy for community oral health beyond the dental school is an appropriate role for dental students. Eighty-four percent agreed or strongly agreed advocacy training should be a required experience in CODA predoctoral education standards (Table 1).

**Table 1:** Dental Student's Attitudes toward, Advocacy (Total *N* = 47).

**Item**	**Strongly Disagree** ***N* (%)**	**Disagree** ***N* (%)**	**Neutral** ***N* (%)**	**Agree** ***N* (%)**	**Strongly Agree** ***N* (%)**
Advocacy for the oral health of my community beyond the dental school and dental clinic is an appropriate role for dental students.	0 (0)	0 (0)	2 (4)	17 (36)	28 (60)
Advocacy training should be a required experience in the ADA-Commission on Dental Accreditation standards for Doctor of Dental Surgery programs.	1 (2)	0 (0)	7 (15)	26 (55)	13 (28)

Respondents reported a low frequency of exposure to legislative and regulatory processes in oral health policy development as 58% reported seldom or no exposure at all to these topics. Overall, most students reported occasional exposure to topics such as community dental health programs, Medicaid and CHIP, child oral health disparities, cultural factors, and access to care. Over half of respondents reported seldom or no exposure to child and family services available in their local and state community (Table 2).

**Table 2:** Frequency of Exposure to Various Topics as part of DDS Didactic Curriculum (Total *N* = 47).

**Item**	**Never** ***N* (%)**	**Seldom** ***N* (%)**	**Sometimes** ***N* (%)**	**Frequently** ***N* (%)**
Legislative and regulatory processes in oral health policy development	7 (15)	20 (43)	19 (40)	1 (2)
Community dental public health programs	1 (2)	2 (4)	26 (55)	18 (38)
Medicaid and CHIP (Children's Health Insurance Program) financing of dental care	2 (4)	8 (17)	21 (45)	16 (34)
Child oral health disparities^*^	0 (0)	5 (11)	23 (50)	18 (39)
Child and family services available in your local and state community	3 (6)	16 (34)	20 (43)	8 (17)
Cultural factors and diversity in health care	0 (0)	6 (13)	23 (49)	18 (38)
Courses in policy, public health, health systems, or similar^*^	3 (7)	14 (30)	20 (43)	9 (20)
Navigating Oral Health Literacy with patients	2 (4)	10 (21)	20 (43)	15 (32)
Issues related to access-to-care for patient populations	4 (9)	7 (15)	22 (47)	14 (30)

* *Not all respondents answered this question (Total N = 46)*.

The questionnaire queried respondents about their experience in advocacy, using a broad definition and range of experiences (Table 3). The most frequent activities were participation in delivery of care to underserved communities through university's mobile dental unit (98%), delivery of free dental services in programs such as Give Kids a Smile Day (90%), dental screenings for Head Start (87%) and promoting school-based sealant programs (75%). An overwhelming majority had never participated in the state dental association grassroots legislative activities nor had contacted legislators related to dental issues (87%). Seventeen percent of respondents reported involvement/contribution to a professional political action committee (PAC) at national or state level.

**Table 3:** Respondent's Personal Advocacy Experiences (Total *N* = 47).

**Item**	**Never participated** ***N* (%)**	**Participated in the last 3 years** ***N* (%)**	**Currently participating** ***N* (%)**
Lobbying at the Statehouse through the ODA & ASDA's Day-at-the-Statehouse Program	38 (81)	7 (15)	2 (4)
Promoting oral health policies at the community/state level	31 (66)	12 (26)	4 (9)
Promoting community water fluoridation programs	28 (60)	14 (30)	5 (11)
Promoting school-based sealant programs	12 (26)	30 (64)	5 (11)
Participation and/or contribution to a Political Action Committee (PAC) (i.e., The American Dental Political Action Committee (ADPAC) of the ADA or state PAC)	39 (83)	6 (13)	2 (4)
Participation in AdvanceOHIO with the ODA (ODA's statewide grassroots advocacy network)	39 (83)	7 (15)	1 (2)
Contacting legislators regarding legislature related to dental-related issues	37 (79)	8 (17)	2 (4)
Contacting policymakers regarding Medicaid and/or CHIP	41 (87)	6 (13)	0 (0)
Donated dental services, Give Kids a Smile Day, or others	5 (11)	29 (62)	13 (28)
Participated in The Ohio State University's Head Start screening program	6 (13)	38 (81)	3 (6)
Working with the Dental H.O.M.E. (Health Outreach Mobile Experience) Coach^*^	1 (2)	33 (72)	12 (26)

* *Not all respondents answered this question (Total N = 46)*.

Most respondents reported that they intend to engage in promotion of oral health policies after graduation at the community/state level and with other professionals. 79% of respondents intend to engage at the community/state level and 89% intend to promote oral health with other professionals. The majority of respondents intend to promote community prevention programs such as community water-fluoridation (91%) and school-based sealant programs (88%) after graduation. Less than a quarter of respondents (21%) had definite intentions to participate and/or contribute to an organized dentistry political action committee. One third of students reported definite intentions to contact legislators related to dental issues or to contact policymakers regarding Medicaid/CHIP in the future. Half of the respondents reported definite intentions to work in an underserved community or accept Medicaid plans. Most respondents (85%) intend to participate in volunteer activities delivering free dental care to underserved or volunteer for committee work in organized dentistry post-graduation (Table 4).

**Table 4:** Intentions for Future Advocacy Involvement Post-Graduation (Total *N* = 47).

**Item**	**Yes** ***N* (%)**	**No** ***N* (%)**	**Undecided** ***N* (%)**
Will promote oral health policies at the community/state level	37 (79)	2 (4)	8 (17)
Will promote oral health policies with other professionals.	42 (89)	1 (2)	4 (9)
Will promote community water fluoridation programs and efforts	43 (91)	0 (0)	4 (9)
Will promote school-based sealant programs	42 (89)	0 (0)	5 (11)
Will participate and/or contribute to a Political Action Committee (PAC) (i.e., The American Dental Political Action Committee (ADPAC) of the ADA or state association PAC)	10 (21)	15 (32)	22 (47)
Will contact legislators regarding legislature related to dental-related issues	16 (34)	13 (28)	18 (38)
Will contact policymakers regarding Medicaid and/or CHIP	14 (30)	15 (32)	18 (38)
Will work in an underserved community	25 (53)	11 (23)	11 (23)
Will join or open a dental practice that accepts Medicaid or Medicaid managed care plan	24 (51)	12 (26)	11 (23)
Will participate in my state dental association & ADA lobbying efforts at the state and/or national level	20 (43)	12 (26)	15 (32)
Will volunteer time and dental services in the form of Free Clinic participation	40 (85)	2 (4)	5 (11)
Will host or participate in dental care initiatives for free services for underserved communities (i.e., Mission of Mercy, Dentistry from the Heart, GKAS)	41 (87)	2 (4)	4 (9)
Will engage in organized dentistry at local, state, or national level (e.g., committee volunteer, etc.)	40 (85)	1 (2)	6 (13)

Further analysis revealed a significant association between advocacy experiences during dental school and intentions for future participation post-graduation. Future intentions were categorized as yes vs. no/undecided. Students who had engaged in lobbying at the statehouse through the Ohio Dental Association (ODA) or the American Student Dental Association during their predoctoral education (current or within past 3 years) were more likely to report definite intentions to contact legislators regarding dental issues (odds ratio = 5.6 with 95% CI (1.17, 26.72), *p* = 0.031) or participate in state dental association/ADA lobbying efforts post-graduation (odds ratio = 17.33 with 95% CI (1.94, 154.64), *p* = 0.011). In addition, students who reported participation or contribution to PAC while in dental school were more likely to express intentions to contribute in future (odds ratio = 11.33 with 95% CI (2.05, 62.77), *p* = 0.005) or contact legislators regarding dental issues post-graduation (odds ratio = 8.7 with 95% CI (1.505, 50.283), *p* = 0.016). Lastly, students who participated in AdvanceOHIO (ODA's statewide grassroots advocacy network) while in dental school were more likely to contribute to dental PACs (odds ratio = 84 with 95% CI (7.59, 929.35), *p* = < 0.001), contact legislators regarding dental issues (odds ratio = 23.33 with 95% CI (2.53, 215.66), *p* = 0.005), contact policymakers regarding Medicaid and/or CHIP (odds ratio = 5.56 with 95% CI (1.11, 27.89), *p* = 0.037), and participate in state dental association and ADA lobbying efforts in the future (odds ratio = 14 with 95% CI (1.55, 126.17), *p* = 0.019). In above statistical results, a larger odds ratio implies stronger effect of advocacy experiences during dental school on the future intention. In particular, the effect of AdvanceOHIO has large odds ratios over 10 for several future intentions for participations, implying explicitly significant association. Some upper bounds of confidence intervals are large because of the large effect size and a certain amount of variation in the sample of size 47.

## Discussion

Advocacy is a broad term and encompasses many educational experiences mandated by CODA. The Ohio State University College of Dentistry engages its students in community-based block rotations, volunteer service opportunities, cultural competency training, and training in basic public health principles. However, there is no formal advocacy curriculum that entails other critical components, such as legislative or public policy advocacy. Despite this, most students had a positive attitude toward, a formalized advocacy-training curriculum and inclusion of such training in predoctoral CODA standards was supported.

Dentists are in a unique and privileged position to advance the oral health of populations through public policy and advocacy. Dentist's high level of specialized knowledge and credibility with policy makers makes their engagement critical for oral health advocacy. However, many dentist lack the broader knowledge, skills, and resources needed to influence the full spectrum of factors affecting oral health [10]. Inclusion of advocacy training in predoctoral education will better prepare dental students for their role as future dentist-advocates and address the gap in knowledge and skillsets needed for effective advocacy.

Decisions made by policy makers at the federal, state and local levels have significant impact on the practice of dentistry and population health, including availability, accessibility and affordability of oral health services [10]. To be effective advocates, dental professionals need to have familiarity with the public policy process, understand the political environment, identify decision makers, and be able to work with grassroots networks and coalitions [10, 16]. For clinicians, these skills may not come naturally when the focus during educational years is on mastering clinical competency. With advocacy training, dental students can gain an understanding of health care system and learn how to affect positive changes within this system [10].

It is clear that advocacy education and practical experience is limited in the predoctoral dental curriculum. In 2018, [17] conducted a needs assessment of Medicaid and health care reform education at University of North Carolina and concluded, “Medicaid and health reform knowledge is poor and scarcely covered in the current curriculum” and curricular improvements were needed at the predoctoral level [17]. Separately, a legislative advocacy exercise was completed with third-year dental students at the University of North Carolina, after which students requested more advocacy exposure throughout the curriculum [18].

Engaging students in advocacy education is important for individuals to develop interest and future goals to become oral health advocates as dentists. Approaches to involve practical advocacy experience for dental students have been described in the literature with promising results. Indiana University involved dental students at an annual event held in collaboration with the Indiana Dental Association and the Children's Dental Health Project to introduce students to the health policy process and to encourage their engagement in advocacy. The day consisted of didactic instructions, small-group activities such as mock legislative presentations, observing Indiana State House deliberations, and meeting with legislators. It was found that 43% of fourth-year dental students who participated in the event reported being “more inclined to become involved with the political process” after participation [10]. The present study found that fourth-year dental students who had exposure to legislative and public policy advocacy were more likely to report intentions to participate in advocacy activities in the future. This finding was similar to a study in medicine where medical students who attended a single legislative advocacy experience felt more likely to contact or meet in person with their legislators about healthcare issues and engage in future advocacy. Additionally, medical students did not agree that their “formal curricula adequately covered legislative healthcare advocacy” [19]. A separate study surveyed pediatric dentists and found that those who received advocacy training as part of their predoctoral dental education or residency program were more willing to engage in advocacy-related activities such as promoting community water fluoridation [20].

Didactic and practical experience with advocacy has been a mandated part of pediatric dentistry specialty education since 2013 [8]. Many advanced education in pediatric dentistry training programs give graduate students legislative experience through participation at the annual American Academy of Pediatric Dentistry's Public Policy Conference in Washington, DC. The conference includes training on current AAPD public policy issues, practical instruction on how to advocate in legislative settings, and congressional visits to representatives of graduate students' home districts. Graduate students attending this conference in 2016 reported gaining valuable skills including “information acquisition about advocacy and public policy in pediatric dentistry; encouragement and motivation for social action at the local level; social network opportunity for peer collaboration and mentorship opportunities; and communication and public speaking” [21]. In addition, residents were more likely to contribute to the AAPD PAC following attendance.

Providing advocacy education and experience in dental school is important to develop this skill set, but providing students an opportunity to attend policy forums and conferences at the state or national level may be costly. Many state dental associations engage in legislative advocacy and may provide members with an opportunity for direct communication with lawmakers without travel to Washington DC or state capitols. Opportunities may also exist for legislative advocacy without travel expense by leveraging teleconference technology. With formal advocacy training, dental students may be better able to identify oral health problems caused by systemic or structural issues that cannot be solved in a dental office and gain the skills to help tackle them. Future research opportunities exist to evaluate the feasibility and efficacy of integrating predoctoral advocacy education programs into the mainstream curriculum.

There are a few limitations to this study. The findings of this study may not be generalizable as the study included only students from one dental school. As with any questionnaire, responses are self-reported and therefore represent a potential for bias. Even though the response rate is considered relatively good for web-administered surveys, [22] the survey did not include all fourth-year dental students, which introduces self-selection bias. Despite these limitations, this study is the first of its kind to look at factors that may lead to dentist's engagement in advocacy after dental school.

## Conclusions

Policies enacted today will have a significant impact on the future of the profession. Dentists are in a unique position to influence legislative proposals, and to do so, they need to acquire appropriate knowledge and skills. There is limited exposure to legislative and public policy advocacy in predoctoral dental education. Dental students with advocacy experience in dental school are more likely to see themselves in an active role as advocates. Therefore, dental education has an opportunity to increase advocacy training in the predoctoral curriculum to support development of a skilled workforce of dentist-advocates for the future.

## Data Availability Statement

The raw data supporting the conclusions of this article will be made available by the authors, without undue reservation.

## Ethics Statement

The studies involving human participants were reviewed and approved by The Ohio State University Office of Responsible Research Practices. Written informed consent for participation was not required for this study in accordance with the national legislation and the institutional requirements.

## Author Contributions

HA and AW contributed to conception, design, and conduct of the study. XG and AN organized the database and performed the statistical analysis. HA and JB wrote the manuscript. All authors contributed to manuscript revision, read, and approved the submitted version.

## Funding

This research and publication was supported by the Ohio State University College of Dentistry.

## Conflict of Interest

The authors declare that the research was conducted in the absence of any commercial or financial relationships that could be construed as a potential conflict of interest.

## Publisher's Note

All claims expressed in this article are solely those of the authors and do not necessarily represent those of their affiliated organizations, or those of the publisher, the editors and the reviewers. Any product that may be evaluated in this article, or claim that may be made by its manufacturer, is not guaranteed or endorsed by the publisher.
